# Influence of different tooth preparation and bonding techniques on the fracture resistance of tooth fragment reattachment

**DOI:** 10.1080/26415275.2021.1952873

**Published:** 2021-07-20

**Authors:** Shaymaa M. Nagi, Sherif M. Khadr

**Affiliations:** a Restorative and Dental Materials Department, Oral and Dental Research division, National Research Centre, Cairo, Egypt; b Conservative Dentistry Department, Faculty of Oral and Dental Medicine, Future University, Egypt

**Keywords:** Fracture resistance, tooth reattachment, bonding, over-contouring, bevel

## Abstract

**Purpose:**

comparing the influence of different tooth preparation and bonding techniques on the fracture resistance of tooth fragment reattachment.

**Materials and method:**

Ninety bovine central incisors were selected. Fifteen teeth act as a control (Group A). Experimental specimens were sectioned at the mesial-incisal proximal edge 3 mm from the incisal edge in a labio-lingual direction at 25degree inclination apically. Experimental specimens were then divided into five groups according to the tooth reattachment techniques utilized; Group B: no tooth preparation + Cured bond + Flowable composite; Group C: no tooth preparation + Uncured bond + Flowable composite; Group D: Bevel + bond + Flowable composite; Group E: Over-contouring + bond + Nanohybrid composite; Group F: Over-contouring + bond + Flowable composite. Specimens were subjected to thermocycling between 5 °C and 55 °C for 500 cycles with 30 sec. dwell time. Fracture strength was evaluated using universal testing machine. Data was analyzed using One-way ANOVA.

**Results:**

There was a statistically significant difference between Group A and all the experimental groups, *p* < .001. Group E showed the highest statistically significant fracture resistance mean value compared to other experimental groups, while the lowest mean value was found in Group B.

**Conclusion:**

Though, none of the tested techniques resulted in fracture resistance similar to that of intact teeth, over-contouring technique with nanohybrid composite application showed better performances compared to the other techniques tested in the current study. Bonding plus flowable resin composite application with no additional tooth preparation and placement of a bevel are not suggested due to the low fracture strength achieved.

## Introduction

Due to the effective caries preventive programs among children and adolescents, it has been expected that in the near future the prevalence of dental trauma might exceed the prevalence of caries and periodontal disease. The most common dental trauma is the coronal fractures of anterior teeth [1].

Regarding the therapy of these dental injuries, several methods were attended throughout the past years, for example veneers and ceramic crowns. However, these procedures tend to sacrifice too much dental hard tissue and have problems related to aesthetic matching of the adjacent and nonrestored teeth [2].

Today, the most conservative treatment with improved aesthetic of such traumatized anterior teeth is achieved by reattachment of the original tooth fragment [3].

The interface between the reattached part and the remaining tooth structure remains the weakest zone in the tooth. Thus, many tooth preparation techniques have been recommended to increase the retention of the reattached fragment. For example, enamel beveling of the fragment and remaining crown, the over-contour technique, internal dentin groove, external chamfer, all of which have their own advantages and disadvantages [2,4].

On the other hand, other studies involve reattachment of the fractured tooth fragment with adhesives or with adhesives and composites without any additional preparation [1,2]. The thickness of the adhesive layer is an important factor for ideal wetting of the substrate and improving the bond strength. It is assumed that reducing the adhesive layer thickness could produce more hermetic bond between the substrates [5].

Thus, this study aimed to measure and compare the effect of different reattachment techniques on the fracture strength of reattached tooth fragments. The tested null hypothesis was that the fracture strength of the tooth fragment reattachment is independent on the different reattachment techniques.

## Materials and methods

### Selected materials

One multimode adhesive system (SingleBond^TM^ Universal) and nano-hybrid universal resin composite (Filtek^TM^ Z250^XT^), and one visible light cured nano-filled flowable resin composite (Filtek^TM^ Supreme Ultra Flowable Restorative) were used in the current study. Materials name, manufacturers and their composition are presented in Table 1.

**Table 1.** Materials names, composition, manufacturer and batch numbers.

Material	Composition	Manufacturer	Batch no
Single bond^TM^ Universal Adhesive	Universal Adhesive: MDP Phosphate Monomer, Dimethacrylate resins, HEMA, VitrebondTM Copolymer, Filler, Ethanol, Water, Initiators, Silane. (pH 2.7)	3M ESPE. (St. Paul, MN 55144-1000, USA)	N489945
Filtek^TM^ Z250 XT Nano-hybrid universal resin composite	BIS-GMA, UDMA, BIS-EMA, PEGDMA, TEGDMA, combination of surface modified zirconia/silica and 20 nm particles. Filler loading 82%weight (60% by volume)	3M ESPE. (St. Paul, MN 55144-1000, USA)	NA28405
Filtek^TM^ Supreme Ultra Flowable Restorative	Bis-GMA, TEGDMA and Procrylat resins. Ytterbium trifluoride filler (0.1 to 5.0 microns), a non-agglomerated/non-aggregated surface-modified 20 nm and 75 nm silica filler, and a surface- modified aggregated zirconia/silica cluster filler (comprised of 20 nm silica and 4 to 11 nm zirconia particles). The aggregate has an average cluster particle size of 0.6 to 10 microns. The inorganic filler 65% by weight and 46% by volume).	3M ESPE. (St. Paul, MN 55144-1000, U.S.A.)	N664994
Meta etchant gel	37% phosphoric acid in water, thickening agent and colorants (pH = 0.5)	Meta Biomed, Germany	MET1906071

MDP: methacryloyloxydecyl dihydrogen phosphate HEMA: Hydroxyethylmethacrylate BIS-GMA: Bisphenol-A-diglycidylmetharylate TEGDMA: Triethyleneglycol dimethacrylate UDMA: Urethane dimethacrylate BIS-EMA: Ethoxylated bisphenol-A glycol dimethacrylate PEGDMA: Polyethylene glycol dimethacrylate.

### Teeth selection

Ninety extracted bovine sound maxillary incisor teeth were utilized in the current study. Teeth were scraped with hand scaler and washed under running tap water to remove any residual tissues and debris [3]. All teeth were examined under magnifying lens (4x) to exclude any teeth with any defects. Teeth were stored at 4 °C immersed in a solution containing 0.2 g sodium azide 100 ml distilled water for a maximum period of one month before being used [6].

### Specimens grouping and specimens’ sectioning

The 90 bovine incisors were divided into two groups: 15 teeth for the control group (**Group A**: Intact sound teeth not subjected to sectioning) and 75 teeth for the experimental groups (**Group B, C, D, E**, and **F (***n* = 15 each)). Sample size calculation was done using R statistical package, version 2.15.2 (26-10-2012). Copyright (C) 2012 - The R Foundation for Statistical Computing. In a one-way ANOVA study, results showed that a total sample size of 15 samples will be adequate to detect a mean difference between study groups with a power of 80% and a two-sided significance level of 5%.

Experimental teeth were sectioned at the mesial-incisal proximal edge 3 mm from the incisal edge in a labio-lingual direction at 25° inclination apically using a diamond disc (Dental Golden S.A.W., Switzerland). Fragments were matched and stored at room temperatures in sterile water for no longer than 48 h. Root of each tooth was embedded in a mass of acrylic resin (Acrostone dental and medical supplier, Egypt) so as to leave only the crown of the tooth exposed. This was done using cylindrical two halves split Teflon mold (2 cm internal diameter and 1 cm height).

### Specimens reattachment procedures

Tooth reattachment procedures were done in the experimental groups as follows:

**Group B** (*n* = 15): Simple re-attachment (no additional tooth fragments preparation was made). The sectioned fragments were reattached using cured bond + flowable composite. Both parts of the tooth were etched for 15 s on dentin and 40 s on enamel with 37% phosphoric acid, after which they were washed thoroughly for 30-40 s and were then blot dried gently with a dry cotton pellet. The adhesive system was applied on both dental fragments using a microbrush and left undisturbed for 10–15 s. The adhesive system was then rubbed for 20 s. The adhesive layer was air thinned using gentle oil free compressed air for 5 s to evaporate the solvent. The adhesive layer was light cured for 10 s according to the manufacturer’s instruction using LED light curing (Elipar S10, 3 M ESPE; USA) unit at intensity 1200 mW/Cm^2,^ without the fragments being in contact. Afterwards a thin layer of the flow composite was applied on both dental fragments, and finally, they were repositioned, with a light finger pressure. Excess material was removed from the buccal and lingual aspect with an applicator. Specimens were light cured for 20 s both from palatal and labial surface. During light curing, some finger pressure was exerted on the incisal dental fragment [1].

**Group C** (*n* = 15): Simple re-attachment (no additional tooth fragments preparation was made), The sectioned fragments were re-attached using uncured bond + flowable composite. The same previously mentioned reattachment procedure as group B was carried out except for that there was omitting of the adhesive system layer curing before the flowable resin composite application. **Group D** (*n* = 15): The tooth preparation technique was modified by preparing a 1.5 mm bevel bucally, at the fracture line on both dental fragments. For this purpose, a 1 mm diameter medium coarse cylindrical diamond bur was used (Oko dent, Germany). This bevel of the two surfaces was etched with 37% phosphoric acid for 30–40 s, then washed thoroughly with water and dried with a dry cotton pellet. Afterwards the adhesive and the flow composite were applied in the same way as in group B. The groove between the two beveled surfaces was filled with flowable composite, followed by light curing for 20 s according to the manufacturer instructions using LED light curing [1].

**Group E** (*n* = 15): Over contouring group, before performing the re-attachment of the fractured tooth fragments, the teeth were prepared on the buccal surface by means of cylindrical diamond finishing bur (W&H diamond burs, Australia) extending 2.5 mm coronally and apically from fracture line at a depth of 0.3 mm [2]. Application of the adhesive system was done as previously mentioned in group B, followed by application of the nanohybrid composite, and light curing for 10 s according to the manufacturer instructions using LED light curing unit.

**Group F** (*n* = 15): Over contouring preparation was done as in group E. Application of the adhesive system was done as previously mentioned in group B, followed by application of flowable composite, then light curing for 20 s according to the manufacturer instructions using LED light curing.

To achieve the perfect repositioning of the dental fragments, a 4x magnifier loupe (Heine Optotechnik GmbH& Co. KG, Germany) was used.

### Aging of the specimens

Reattached teeth were finished and polished with the flexible polishing disk (Sof-Lex, 3 M ESPE, USA) and were then stored in artificial saliva for 48 h in an incubator adjusted at 37 °C, then specimens were subjected to thermo-cycling between 5 °C and 55 °C (±5 °C) for 500 cycles with 30 s dwell time [7].

### Fracture strength of restored teeth

**Figure 1.**
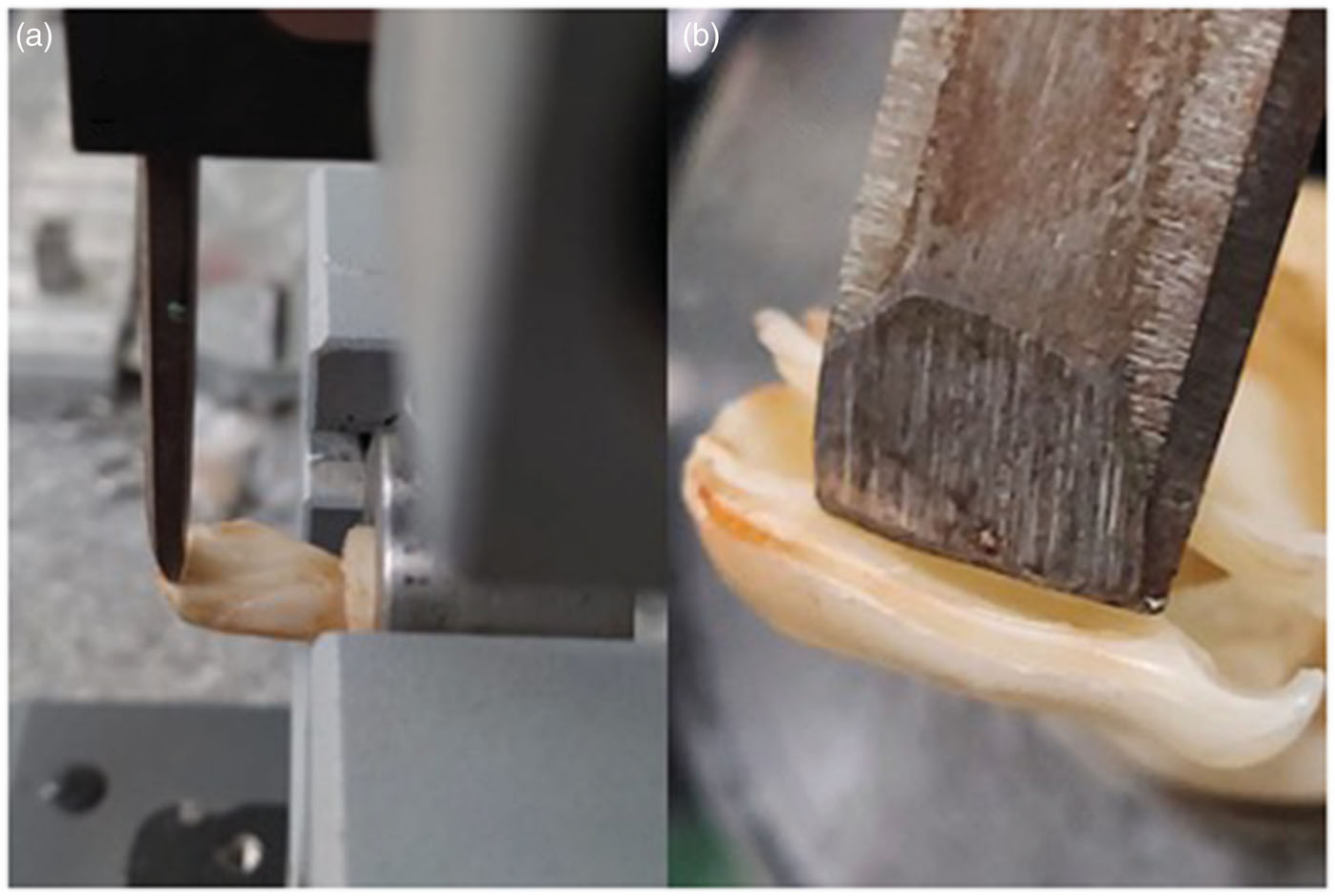
(a) Specimen attached to a universal testing machine; (b) a chisel delivers the force 2 mm from the incisal edge.

The specimens were mounted on a custom-made fixture for determination of fracture strength using universal testing machine. A chisel of 0.5 mm in cross section was used to deliver the force so that contact was achieved 2 mm from the incisal edge with a crosshead speed of 1 mm per minute (Figure 1(a,b)). The load required to dettach each specimen’s fragment was recorded in kilogram forcing units (kgf) [8].

Finally, an analysis was performed on the failure mode using a stereomicroscope (50x) (Nikon, SMZ-2 Japan) after loading the restored teeth, categorizing them into [9]:

- Adhesive failure: when the tooth fractured along the bonded interface.
- Mixed failure: when it involved both the bonded interface and other portions of dental substance.

### Statistical analysis

The mean and standard deviation values were calculated for each group. Data were explored for normality using Kolmogorov–Smirnov and Shapiro–Wilk tests, data showed parametric (normal) distribution. One-way ANOVA followed by Tukey *post hoc* test was used to compare between more than two groups in non-related samples. The significance level was set at *p < .001*. Statistical analysis was performed with IBM^®^ SPSS^®^ Statistics Version 20 for Windows.

## Results

Table 2 shows the mean, standard deviation (SD) values of fracture strength (KgF) of different groups and the fracture strength recovery (%) of each group was calculated.

**Table 2.** The mean, standard deviation (SD) values of fracture strength of different groups.

Variables	Fracture strength (KgF)		
	Mean	SD	Fracture strength recovery %**
**Group A: Intact tooth**	76.92^a^	3.74	100%
**Group B: Cured bond + Flowable composite**	32.08^c^	4.76	41.7%
**Group C: Uncured bond + Flowable composite**	39.12^c^	4.51	50.85%
**Group D: Bevel + bond + Flowable composite.**	37.25^c^	5.20	48.42%
**Group E: Over contouring + bond + Nanohybrid composite**	52.65^b^	2.74	68.44 %
**Group F: Over contouring + bond + flowable composite**	34.83^c^	3.52	45.28%
*p*-value	<0.001*		

*significant (*p* < .05) ** Fracture strength recovery was calculated based on the mean and SD of the fracture strength of sound teeth (100%).

There was a statistically significant difference between (Group A), (Group B), (Group C), (Group D), (Group E) and (Group F) where (*p* < .001). A statistically significant difference was found between (Group A) and each of Group B, (Group C), (Group D), (Group E) and (Group F) where (*p* < .001). Also, a statistically significant difference was found between (Group E) and each of (Group B), (Group C), (Group D) and (Group F) where (*p* < .001). While no statistically significant difference was found between any other groups. The highest mean value was found in Group A (Intact tooth), while the lowest mean value was found in Group B (Cured bond + Flowable composite).

Fracture strength recovery (%) was 41.7%, 50.85%, 48.42%, 68.44%, 45.28% for groups B, C, D, E and F, respectively.

**Figure 2.**
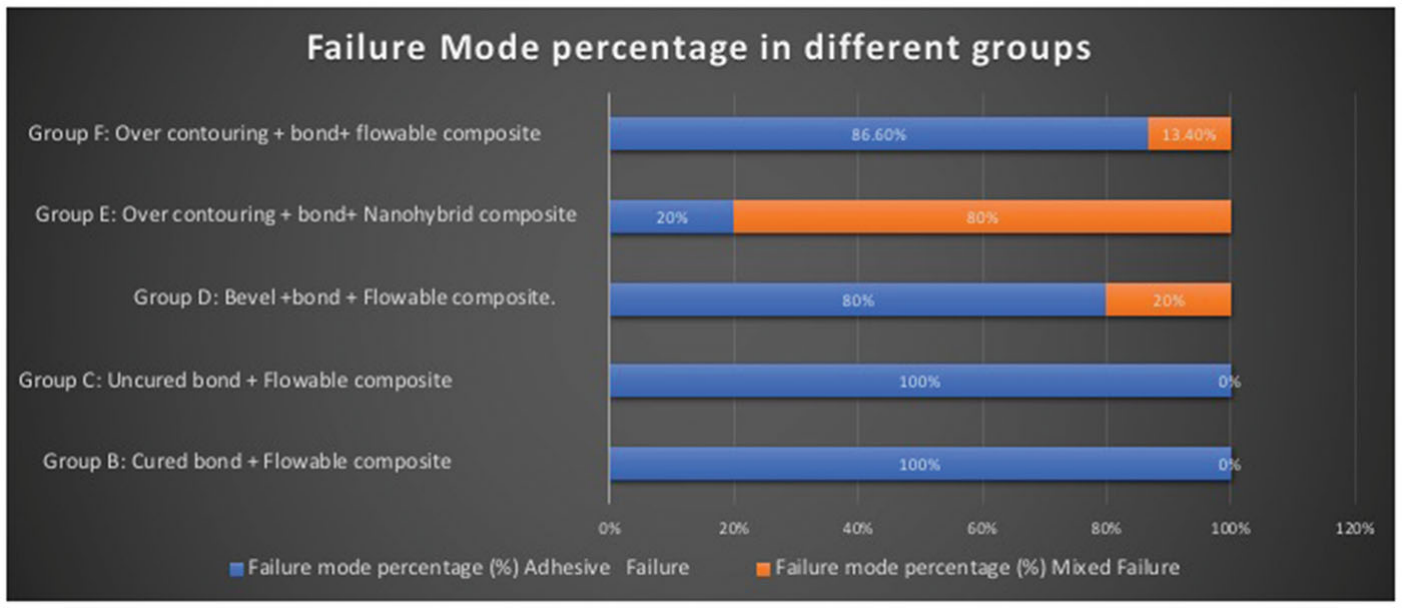
Failure mode percentage in different groups.

Failure mode analysis showed that Groups A fractures was extending below the cementoenamel junction. Group E showed more than 80% mixed failure that always involved the apical tooth structure at the bonded interface in addition to previously intact portions of the tooth. While in groups B, C, D, and F the adhesive failure at the bonded interface (the fracture only involved the previously reattached fragment and never the structure of the tooth) was the most shown, (Figure 2).

## Discussion

One of the unfortunate consequences of trauma, is the fracture of anterior teeth. Fractured anterior teeth has many tragic effects: not only because of damage to dentition but also it has psychological effects on the patients [2,10].

Many restorative treatment alternatives have been used in the past for restoration of fractured teeth depending on the site of fracture, i.e. resin crowns, stainless steel crowns, orthodontic bands, pin-retained resin, porcelain jacket crowns, porcelain bonded crowns and resin composite restorations. All these options are consistent; nevertheless, these procedures are not conservative, have problems in obtaining tooth color, translucency and contour to harmonize it to the remaining crown portion. Moreover, these techniques are also time-consuming and high cost [11].

Taking into account the disadvantages shown by the conventional restorative techniques, it was proposed to restore the fractured tooth with the dental fragment [11]. This tooth reattachment technique has many benefits comparing to the other techniques. It is considered the most conservative procedure, with maximum aesthetic recovery as the tooth color, contour, surface texture, and translucency are the same as that of the natural tooth. Moreover, there is similar color stability and wear as the other teeth [2,10].

In the current study, bovine incisors were utilized instead of human teeth as they were easier to be collected in sound state. Several studies counted bovine teeth as viable substitutes for human teeth. As comparative morphological and histochemical studies showed that all mammalian teeth are fundamentally alike [12–14]. Studies found nearly same diameter and number of coronal dentin tubules and the assessment of enamel morphology after acid etching showed that the bovine and human enamel had obviously similar aspects [12,13]. Thus, it is expected that the fracture strength test gives similar results in both bovine and human teeth [14].

The experimental specimens in the current study were sectioned at 3 mm from incisal edge at a 25° inclination apically in a facio-lingual direction using a diamond disc. This was done to simulate most of traumatized incisors fracture which is in an oblique manner from labial to lingual aspects with the fracture line proceeding in an apical direction. This is considered as a critical fracture pattern that shows low resistance to the functional forces. This also happens when fractures are nearly perpendicular to long axis of the tooth [7].

In the current study, a diamond disc was used to section the incisal edge of each specimen. This was considered a limitation of this study. As the obtained fractured surface from the disc differs from natural fracture, disc sectioned specimens have a smear layer that is not present on a natural fracture. Moreover, the natural fractured surface frequently happens parallel to the main direction of the enamel prisms, while the direction of the sectioned surface is determined by the orientation of the cut. In addition, a naturally occurring fracture gives fragments with a good fitting [15], while disc cut specimens resulted in imperfect approximation of the tooth and the fragment. Thus, the results found in current study might be an underestimation of what could be attained clinically using the tested techniques. Although all the previously mentioned limitation of the specimens sectioning procedure utilized in the current study, as the sectioned fragment confirms consistent, standardized and repeatable situation that was extremely essential for an *in vitro* study [16].

Teeth and dental fragments were kept in a saline solution after sectioning to avoid dehydration of the dental surfaces that might negatively affect bonding to the reattached fragments [17,18].

Results of the current study revealed that reattachment of the fractured fragment in the four experimental groups had statistically significant lower fracture strength compared to the control group. The literature reinforces this finding irrespective of the materials and techniques utilized [1,19].

In groups B and C, tooth fragments reattachment was done using the adhesive in combination with a thin layer of flow composite. In group C, the adhesive layer was not cured before application of the flowable composite layer. By forcing the flowable composite into the uncured adhesive, the adhesive performs like a wetting agent [5] filling only the irregularities in the surface of the tooth fragments. On the other hand, curing the adhesive layer before the flowable composite application as in group B, gives the adhesive layer more thickness compared to the uncured adhesive layer in group C. Although at the reattachment of the fragments pressure was exerted, cured adhesive layer still have some thickness, making the intermediary layer slightly thicker. Maybe this is the reason for the higher non statistically significant in the fracture resistance for group C compared to group B.

Comparing the teeth restored only with adhesive and flowable composite (group B and C) with the bevelled teeth bonded with flow composite (group D); in the latter group, a better union was observed. This might be due to adding a bevel increases the acid etched enamel surface area; thus provide a better micromechanical retention [1,20–22]. In spite of this, the difference found in the current study was not statistically significant. This might be due to that the mechanical strength and/or the elasticity of the flow composite declines as the contact surface increases [1].

Regarding comparing over the counter tooth preparation groups: group F (Over contouring preparation + bond + flowable resin composite) showed statistically significant lower fracture strength compared to group E (Over contouring + bond + nanohybrid resin composite). Filtek^TM^ Supreme Ultra Flowable resin composite used in group F, has low filler loading (65% by weight and 46% by volume) which decrease its mechanical properties and increase its polymerization shrinkage compared to nanohybrid resin composite utilized in group E. Thus, this could lead to more debonding of the material during polymerization and lower its strength [23].

Over contouring tooth preparation + bond + nanohybrid resin composite (Group E) showed the highest mean fracture resistance compared to other groups. In this tooth technique preparation, nanohybrid composite (Filtek^TM^ Z250 XT) was utilized to reattach the tooth fragments. Its high fracture resistance might be related to its high filler loading 82% by weight (60% by volume). Filler loading is a fundamental factor in load-bearing ability. Increased filler loading allows excellent strength and durability to withstand the stresses of the dentition and the oral cavity [24,25]. This result was supported by failure mode analysis, where an increased mixed failure percentage (80%) (Failure include the bonded interface plus cohesive failure in the tooth substrate) was shown in group E. Cohesive failure in the tooth structure confirms that this tooth fragment reattachment technique improve the fracture strength due to increasing the stiffness of tooth restoration complex [26,27].

Finally, in the present study the null hypothesis that the fracture strength of the tooth fragment reattachment is independent on the different reattachment techniques was rejected as there was statistically significant difference between over the counter tooth preparation + bond + nanohybrid resin composite and the other tested tooth fragment reattachment techniques.

## Conclusions

According to the limitations of the current study, it could be concluded that although, none of the tested techniques resulted in fracture resistance similar to that of intact teeth:

- Over-contouring technique with nanohybrid resin composite application showed better performances compared to the other techniques tested in the current study.
- Bonding plus flowable resin composite application with no additional preparation and placement of a bevel are not suggested due to the low fracture strength achieved.
